# Distinctive physiological and molecular responses of foxtail millet and maize to nicosulfuron

**DOI:** 10.3389/fpls.2023.1308584

**Published:** 2024-01-16

**Authors:** Boyu Lu, Ru Meng, Yiru Wang, Wei Xiong, Yuchao Ma, Peng Gao, Jianhong Ren, Liguang Zhang, Zhihai Zhao, Guangyu Fan, Yinyuan Wen, Xiangyang Yuan

**Affiliations:** ^1^ State Key Laboratory of Sustainable Dryland Agriculture (in preparation), Shanxi Agricultural University, Taiyuan, Shanxi, China; ^2^ College of Life Sciences, Shanxi Agricultural University, Taigu, Shanxi, China; ^3^ Institute of Millet, Zhangjiakou Academy of Agricultural Science, Zhangjiakou, China

**Keywords:** foxtail millet, maize, nicosulfuron, acetolactate synthase, detoxification enzymes, gene expression

## Abstract

**Introduction:**

Nicosulfuron is the leading acetolactate synthase inhibitor herbicide product, and widely used to control gramineous weeds. Here, we investigated the metabolic process of nicosulfuron into foxtail millet and maize, in order to clarify the mechanism of the difference in sensitivity of foxtail millet and maize to nicosulfuron from the perspective of physiological metabolism and provide a theoretical basis for the breeding of nicosulfuron-resistant foxtail millet varieties.

**Methods:**

We treated foxtail millet (Zhangzagu 10, Jingu 21) and maize (Nongda 108, Ditian 8) with various doses of nicosulfuron in both pot and field experiments. The malonaldehyde (MDA) content, target enzymes, detoxification enzymes, and antioxidant enzymes, as well as related gene expression levels in the leaf tissues of foxtail millet and maize were measured, and the yield was determined after maturity.

**Results:**

The results showed that the recommended dose of nicosulfuron caused Zhangzagu 10 and Jingu 21 to fail to harvest; the yield of the sensitive maize variety (Ditian 8) decreased by 37.09%, whereas that of the resistant maize variety (Nongda 108) did not decrease. Nicosulfuron stress increased the CYP450 enzyme activity, MDA content, and antioxidant enzyme activity of foxtail millet and maize, reduced the acetolactate synthase (ALS) activity and ALS gene expression of foxtail millet and Ditian 8, and reduced the glutathione S-transferase (GST) activity and GST gene expression of foxtail millet. In conclusion, target enzymes, detoxification enzymes, and antioxidant enzymes were involved in the detoxification metabolism of nicosulfuron in plants. ALS and GST are the main factors responsible for the metabolic differences among foxtail millet, sensitive maize varieties, and resistant maize varieties.

**Discussion:**

These findings offer valuable insights for exploring the target resistance (TSR) and non-target resistance (NTSR) mechanisms in foxtail millet under herbicide stress and provides theoretical basis for future research of develop foxtail millet germplasm with diverse herbicide resistance traits.

## 1 Introduction

In a crop production system, weeds compete for the same resources as crops, limiting crop productivity. Globally, a yield loss of up to 40% has been reported due to the resilience and persistence of such non-crop plants (Sharma et al., 2021). Herbicides for weed control generally provide rapid action, are convenient to use, and are thus integral to current and future agricultural practices (Ma et al., 2021). There are many kinds of herbicides, among which sulfonylureas are an important class, as they are widely used to control a series of weeds and gramineous plants, especially in grains (Sarmah and Sabadie, 2002).

Nicosulfuron is a sulfonylurea herbicide and the leading acetolactate synthase (ALS EC 4.1.3.18) inhibitor herbicide product worldwide. The characteristics of nicosulfuron include a wide herbicidal spectrum, high activity, low field use, and good herbicidal effects. It is widely used to control gramineous weeds in maize production (Choe and Williams, 2020). In recent years, germplasm resistant to nicosulfuron herbicides has been created in wheat, transgenic soybean, rice and other major grain and oil crops by using biotechnology, and has been successively applied to weed control in field (Zhang et al., 2019; Zhang et al., 2021a; Zheng et al., 2022). However, Wang et al. (2022a) study showed that the photosynthesis of beet was significantly inhibited under nicosulfuron stress, and the antioxidant reduction system was damaged, ultimately leading to plant death. Meanwhile, nicosulfuron is not safe for sorghum, causing serious yield loss, which shows that its safety to different plants is different (Bowman et al., 2021). As we all known, nicosulfuron impedes the biosynthesis of the branched-chain amino acids leucine, iso-leucine, and valine by inhibiting the activity of ALS, thereby disrupting the biosynthesis of proteins and eventually leading to most plant death (Huang et al., 2019). Whereas, the detoxification process of nicosulfuron after entering the plant has also been implicated in the mechanism of plant death. Therefore, we speculate that the reason for the safety differences of nicosulfuron to different crops may be related to the detoxification metabolism of herbicides.

The mechanisms of plant resistance to ALS herbicides are mainly divided into target resistance (TSR) and non-target resistance (NTSR) (Délye, 2013). ALS enzyme activity is higher in resistant plants than in sensitive plants for ALS inhibitor herbicides, and this may be the factor that enables the said resistance (Chodová and Mikulka, 2018). Domínguez-Mendez et al. suggested that the high activity of ALS is the main reason for the resistance of imidazolinone-resistant wheat varieties (Domínguez-Mendez et al., 2017). Huang et al. (2021) also found that the ALS activity of resistant green millet was 17.8–27.6 times that of sensitive strains under I_50_ (herbicide dose required to inhibit ALS activity by 50%). In addition, the overexpression of the *ALS* gene has an important relationship with plant resistance. After the application of ALS inhibitor herbicides to plants, target proteins are overexpressed, and a large amount of ALS is synthesized to replace the enzymes passivated by the herbicides. Maintaining the basic life needs and normal physiological activities of plants leads to the gradual emergence of resistance (Sen et al., 2021). Overexpression of the *ALS* gene has been shown to lead to herbicide resistance in barnyard grass and maize (Iwakami et al., 2012; Wang, 2018).

NTSR mechanisms are increasingly considered an important method for weed resistance to ALS inhibitor herbicides (Délye, 2013). NTSR mainly refers to the physiological metabolic detoxification process of herbicides through non-target pathways and the process of limiting the ability of herbicides to reach their target sites so that they lose activity, which can help to achieve detoxification (Duhoux et al., 2015). After the herbicide enters the plant, there are three main stages to the metabolic process. First, the herbicide is cleared and metabolized by the oxidation, reduction, and hydrolysis reactions involved in the cytochrome P450 (CYP450) oxidase system. Subsequently, both glutathione S-transferase (GST) and glycosyltransferase are involved in the conjugation of glutathione, carbohydrates, and amino acids. Finally, the ABC transporter transports the exogenous herbicide from the cell to the vacuole, thereby reducing phytotoxicity. Unmetabolized herbicides will destroy membrane lipid peroxidation, promote the production of superoxide anion, hydrogen peroxide, and malondialdehyde, and damage the balance of production and scavenging of reactive oxygen species (ROS) (Liu et al., 2018). The main metabolic enzymes involved include CYP450, superoxide dismutase, and glutelin reductase (Duhoux et al., 2015). CYP450-mediated enhanced herbicide metabolism is one of the most important NTSR mechanisms. Liu et al. (2018) identified the *P450* gene with nicosulfuron tolerance in maize. Wang et al. (2022b) also confirmed that green millet CYP450 plays an important role in herbicide metabolism. Similarly, plants respond to abiotic stress by increasing GST activity, and the overexpression of specific GST isozymes in transgenic plants enhances plant tolerance to herbicides and oxidative stress (Cummins et al., 2013; Singh et al., 2015). Wu et al. (2022) compared the physiological changes of resistant and sensitive maize varieties under nicosulfuron treatment and found that the expression of antioxidant enzymes and related genes in resistant varieties was significantly higher or upregulated compared with the sensitive varieties. Therefore, resistant maize can alleviate the toxic effects of nicosulfuron on plants by scavenging ROS.

Foxtail millet (*Setaria italica* L.) is a new C_4_ model plant that is drought-resistant, barren-tolerant, and nutrient rich. The high and stable yield of foxtail millet, however, is seriously limited by the grass in foxtail millet fields, foxtail millet cultivation has previously relied on artificial thinning and weeding, which is time-consuming and restricts intensive and large-scale production (Ma et al., 2021). There are few herbicides registered in foxtail millet fields, so chemical weeding is particularly important. The weed populations in foxtail millet fields and maize fields are similar. Nicosulfuron can effectively control *Digitaria sanguinalis*, *Echinochloa crusgalli*, *Setaria virid*is, etc., and is safe for maize fields. However, the recommended dose of nicosulfuron causes serious damage to foxtail millet (Huang et al., 2005). Our previous studies elucidated the photosynthetic physiological mechanism associated with the differences in sensitivity between foxtail millet and maize to nicosulfuron from the perspective of photosynthetic fluorescence (Gao et al., 2021). We hypothesized that the differences in sensitivity to nicosulfuron may be related to the activity of its target enzyme, ALS, detoxification enzymes, and antioxidant capacity. The main aims of this study were to (i) explore the safety of different doses of nicosulfuron on foxtail millet and maize, and (ii) further clarify the differences in the TSR and NTSR mechanisms of resistance to nicosulfuron between these two crops. The results will provide a theoretical reference to improve our in-depth understanding of foxtail millet herbicide resistance mechanisms and varietal improvement.

## 2 Materials and methods

### 2.1 Experimental materials

Nicosulfuron-resistant maize variety Nongda 108 was provided by Shandong Denghai Huayu Seed Industry Co. Ltd., and the nicosulfuron-sensitive maize variety Ditian 8 was provided by the Maize Institute, Shanxi Agricultural University. The nicosulfuron-sensitive foxtail millet varieties Zhangzagu 10 and Jingu 21 were provided by the Zhangjiakou Academy of Agricultural Sciences, Hebei Province, and the Economic Crops Research Institute of Shanxi Agricultural University, respectively. A 4% nicosulfuron dispersible oil suspension (PD20096919) was provided by Langfang Pesticide Pilot Plant of the Plant Protection Institute, Chinese Academy of Agricultural Sciences.

### 2.2 Experimental design

In 2021, a field experiment was conducted at the Shanxi Agricultural University farm in China. The soil was loam (carbonate brown soil), and the basic physical and chemical properties were total phosphorus 0.769 g/kg, total potassium 22.81 g/kg, total nitrogen 1.037 g/kg, available potassium 103.6 mg/kg, alkaline hydrolyzable nitrogen 32.19 mg/kg, available phosphorus 9.28 mg/kg, organic matter 20.74 g/kg, and pH 8.72. Briefly, 750 kg of compound fertilizer was applied to each hectare of field before sowing. The experiment was arranged in a 2-factor randomized complete block design with four varieties, five nicosulfuron dosages, and triplicate plots (**Table 1**). The area of each plot of foxtail millet and maize was 6 × 5 m^2^. When the plants had grown to the 3–5 leaf stage, the seedlings were treated with different dosages of nicosulfuron. The applications were performed using a laboratory pot sprayer equipped with a nozzle calibrated to deliver 450 L/ha.

**Table 1:** Dosages of nicosulfuron applied to foxtail millet and maize.

Varieties	CK g/hm^2^	1/8 X g/hm^2^	1/4 X g/hm^2^	1/2 X g/hm^2^	1 X (recommended dose) g/hm^2^	2 X g/hm^2^
Zhangzagu 10	0	7.5	15	30	60	—
Jingu 21	0	7.5	15	30	60	—
Ditian 8	0	7.5	15	30	60	—
Nongda 108	0	—	15	30	60	120

—, not sprayed with nicosulfuron treatment.

The pot experiment was conducted at the Crop Chemistry Control Center of Shanxi Agricultural University, China, in 2021. The experiment adopted a randomized complete block design, and each treatment was repeated three times with a total of five dosage treatments (**Table 1**). Foxtail millet and maize seeds were sown in 11 × 11.5 cm nutrient pots with substrates. The pots were placed in an artificial climate incubator with a photoperiod of 16/8 h (light/dark), temperatures of 25/18°C (light/dark), illumination intensity of 12,000 xl, and relative humidity of 70%–80%. At the three-leaf stage, five test doses of nicosulfuron were applied using laboratory pot sprayers, and the spraying method was the same as that in the field experiments.

### 2.3 Yield and yield components

After field harvest, the ear length, ear weight, and ear grain weight were measured using a ruler and a 10,000 analytical balance (Mettler-Toledo, LLC, Shanghai, China). These indicators were performed in ten technical and biological replicates.

### 2.4 ALS, GST, and CYP450 activities

Seedlings of foxtail millet and maize ​were collected at the three-leaf stage, immediately frozen in liquid nitrogen, and stored at -80°C. Each treatment was repeated three times. The ALS extraction and herbicide inhibition assays were conducted according to previous studies (Liu et al., 2018). The ALS activity was determined colorimetrically (530 nm) using a UV spectrophotometer (Thermo Scientific) by measuring acetoin production. GST activity was determined using the method of Vuković et al. (2022). The reaction mixture consisted of 2 mM GSH, 1 mM CDNB, 1 mM EDTA, and 50 µL of protein extract in 100 mM phosphate buffer (pH 6.5), in a final volume of 1.5 mL. The increase in absorbance was recorded at 340 nm for 2 min every 15 s. GST activity was calculated using a molar extinction coefficient of glutathione-1-chloro-2,4-dinitrobenzene conjugate (ϵ = 9.6 mM/cm). The determination of CYP450 activity was described by Li et al. (2003), which is represented by the fluorescence of the 7-hydroxycoumarin product at 460 nm while exciting at 360 nm.

### 2.5 Gene expression

The fresh leaves of maize and foxtail millet were collected at 1, 3, and 5 d after spraying, respectively, quick-frozen with liquid nitrogen, and then stored at −80°C until the determination of *ALS* and *GST* gene expression. The *ALS* and *GST* gene sequences are available at www.maizegdb.org and www.ncbi.nlm.nih.gov/. Primer Premier 5 was used to design gene primers according to the gene sequences in the database, and the primer sequences were compared in the NCBI database to ensure their specificity. The internal reference gene for the target genes *SiALS* and *SiGST* in foxtail millet is *SiACTIN*, and the internal reference gene for the target genes *ZmALS1*, *ZmALS2*, and *ZmGST* in maize is *ZmEF1α* (**Table 2**). Total RNA was extracted from 100 mg fresh leaves of maize and foxtail millet using Trizol reagent (TransGen Biotech, ER501-01-V2). First strand cDNA synthesis was performed using 1 μg of total RNA from each sample with reverse transcriptase (ABM, G592). The RT-qPCR program was carried out by setting an appropriate Tm temperature using real-time fluorescence quantitative PCR instrument (American Bole Bio-Rad CFX96). The relative expression level of the target gene was calculated by using the 2^-ΔΔct^ method. The experiment was performed with three technical and biological replicates.

**Table 2:** Primer sequences.

Gene Name	Gene ID	Primer Name	Sequence	base length
*SiALS*	XM_004952503.3	SiALS-F	ACACCTACAAGCGTCCAAGG	20
		SiALS-R	GGAAGCTACCATCCCCATCG	20
*SiACTIN*	XM_004971314.4	SiACTIN-F	TGCTCAGTGGAGGCTCAACA	20
		SiACTIN-R	CCAGACACTGTACTTGCGCTC	21
*ZmALS1*	NM_001158289.2	ZmALS1-F	ATGGTGATGGCAGGACTGTG	20
		ZmALS1-R	CTTGTACTAGCAGGGAGGCG	20
*ZmALS2*	NM_001148702.2	ZmALS2-F	GCACTGCCTACCTGCCTATC	20
		ZmALS2-R	CACACAGGGTTGGATGCTACT	21
*ZmEF1α*	XM_020543848.2	ZmEF1α-F	TGGGCCTACTGGTCTTACTACTGA	24
		ZmEF1α-R	ACATACCCACGCTTCAGATCCT	22
*SiGST*	XM_004970008.2	SiGST-F	TGGTGACTTGTGCATCTGGG	20
		SiGST-R	TAGCCTCCACCTCCATCCAA	20
*ZmGST*	NM_001111942.1	ZmGST-F	CGGTGACTTGTACCTCTTCGAATC	24
		ZmGST-R	ATCCACCATTGCTGCCTCC	19

### 2.6 MDA content

MDA was determined using thiobarbituric acid (TBA). Fresh foxtail millet and maize leaf (0.4 g) were homogenized with 5 mL of 0.1% trichloroacetic acid (TCA) in pots. Then, 5 mL of 0.5% TBA was added and mixed well in a glass tube. The reaction mixture solution was boiled for 15 min, cooled quickly, and centrifuged for 15 min at 3000 *× g* at 4°C. The absorbance of the supernatant was then measured at 532 and 600 nm, respectively (Gao, 2006). This indicator was performed in three technical and biological replicates.

### 2.7 Antioxidant enzyme activities

Fresh foxtail millet and maize leaf (0.1 g) in pots were homogenized in 2 mL of 0.05 mol L^-1^ phosphate buffer (pH 7.8) and centrifuged at 12000 *× g* for 15 min at 4°C. The supernatant was extracted for SOD, POD, and CAT activities. SOD activity (EC 1.15.1.1) was determined using the nitro blue tetrazolium (NBT) method. One unit of enzyme activity (U) was defined as the amount of enzyme required to inhibit 50% of the initial reduction of NBT under light conditions. POD activity (EC 1.11.1.7) was determined according to the Guaiacol method. It was measured using the changes in the absorbance of the reaction solution at 470 nm for 3 min. CAT activity (EC 1.11.1.6) was determined using the ultraviolet absorption method. The absorbance was measured at 240 nm and recorded every 30 s for 3 min (Gao, 2006). These indicators were performed in three technical and biological replicates.

### 2.8 Statistical analysis

All data are presented as the mean ± standard error (SE). Statistical data were analyzed using SPSS Statistics software (version 21.0; SPSS, Chicago, IL, USA). Mapping was conducted with Origin 2021. One-way analysis of variance (ANOVA) with Duncan’s multiple range test was used to determine the significant differences among the treatments and varieties at a significance level of *P* ≤ 0.05.

## 3 Results

### 3.1 Effects of nicosulfuron on the yield and yield components of foxtail millet and maize

As the dose of nicosulfuron increased, the ear length, ear weight, grain weight, and yield of foxtail millet showed a downward trend, and the minimum values were recorded with the 1/2X dose. Compared with the control, the yield of Zhangzagu 10 and Jingu 21 decreased by 27.72% and 51.09% under 1/2X treatment, respectively, and the plants died with the 1X dose (**Table 3**). The yield traits of Ditian 8 also showed a downward trend, reaching their minimum values with the 1X dose, and the yield was reduced by 37.09% when compared with the control. Nongda 108 was not affected by nicosulfuron (**Table 4**).

**Table 3:** Effects of nicosulfuron on yield characteristics of foxtail millet in the field.

Varieties	Treatments	Ear length (cm)	Ear weight (g)	1000 grain weight (g)	Yield (t/hm^2^)
Zhangzagu 10	CK	31.74 ± 0.86a	18.88 ± 2.83a	2.92 ± 0.04a	5.75 ± 0.37a
	1/8X	31.32 ± 0.71a	18.41 ± 2.02a	2.78 ± 0.02a	5.64 ± 0.15a
	1/4X	30.06 ± 0.63b	15.95 ± 2.95b	2.62 ± 0.02b	5.18 ± 0.24b
	1/2X	26.16 ± 0.72c	12.26 ± 0.69c	2.41 ± 0.03c	4.15 ± 0.24c
	1X	—	—	—	—
Jingu 21	CK	27.72 ± 0.76a	47.24 ± 3.49a	3.13 ± 0.06a	5.01 ± 0.13a
	1/8X	26.44 ± 1.36a	43.71 ± 3.26a	3.07 ± 0.03a	4.79 ± 0.28b
	1/4X	25.86 ± 1.36b	31.55 ± 1.96b	2.95 ± 0.02b	3.41 ± 0.12c
	1/2X	23.38 ± 1.17c	23.09 ± 1.16c	2.77 ± 0.04c	2.45 ± 0.13d
	1X	—	—	—	—

Data represent means ± standard deviations. For the different nicosulfuron dosages of the same variety, values not displaying the same letter are significantly different (P < 0.05).

—, indicates that the foxtail millet was seriously damaged and failed to harvest.

**Table 4:** Effects of nicosulfuron on yield characteristics of maize in the field.

Varieties	Treatments	Ear length (cm)	Ear weight (g)	100 grain weight (g)	Yield (t/hm^2^)
Ditian 8	CK	17.58 ± 0.15a	120.66 ± 4.11a	15.05 ± 9.18a	6.93 ± 0.42a
	1/8X	17.36 ± 0.43b	125.91 ± 4.25b	14.94 ± 5.51b	6.68 ± 0.35a
	1/4X	15.81 ± 0.27c	100.09 ± 3.98c	13.84 ± 1.93c	5.66 ± 0.28b
	1/2X	15.64 ± 1.59c	86.52 ± 2.49d	13.82 ± 3.56c	5.47 ± 0.14b
	1X	13.51 ± 0.21d	75.92 ± 1.29e	12.97 ± 2.89d	4.36 ± 0.06c
Nongda 108	CK	24.32 ± 0.35a	339.07 ± 11.94a	39.01 ± 0.78a	8.06 ± 0.54a
	1/4X	25.41 ± 2.09a	352.96 ± 14.68a	37.94 ± 4.67a	7.96 ± 0.89a
	1/2X	24.45 ± 0.44a	345.09 ± 8.51a	37.21 ± 0.97a	7.89 ± 0.27a
	1X	25.74 ± 0.95a	358.81 ± 10.67a	36.81 ± 2.13a	8.11 ± 0.42a
	2X	25.75 ± 0.78a	369.19 ± 9.54a	36.07 ± 1.74a	7.94 ± 0.21a

Data represent means ± standard deviations. For the different nicosulfuron dosages of the same variety, values not displaying the same letter are significantly different (P < 0.05).

### 3.2 Effects of nicosulfuron on the ALS activity of foxtail millet and maize

**Figure 1:**
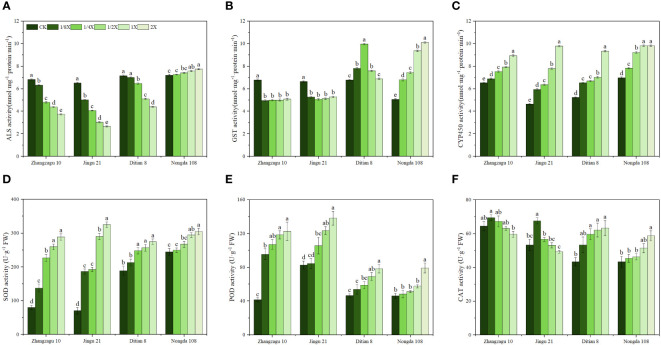
Effects of nicosulfuron on the physiological indices of foxtail millet and maize at 5 d after treatment. **(A)** ALS, **(B)** GST, **(C)** CYP450, **(D)** SOD, **(E)** POD, and **(F)** CAT activities. Data are presented as the mean ± SE (n = 6). Different letters indicate statistically significant differences at *P* < 0.05.

The effects of nicosulfuron on ALS activity in foxtail millet and maize exhibited various trends. With the increase in nicosulfuron, the ALS activity of sensitive Zhangzagu 10, Jingu 21, and Ditian 8 showed a decreasing trend, and the ALS activity of resistant Nongda 108 showed an increasing trend. The ALS activity of foxtail millet was lower than that of maize without adding nicosulfuron. The inhibitory effect of spraying nicosulfuron on ALS activity of foxtail millet was significantly higher than that of maize, and the difference between different treatments was significant. The ALS activity of Zhangzhagu 10 and Jingu 21 decreased sharply at 1/4 and 1/8 doses, respectively, and the same phenomenon occurred in Ditian 8 at 1/2X dose. Under the recommended dose, the ALS activity of Zhangzhagu 10, Jingu 21, and Ditian 8 decreased by 59.28%, 45.21%, and 38.54%, respectively, compared with the control, while that of Nongda 108 increased by 7.6%. It is speculated that maintaining high and stable ALS activity may be one of the reasons for the difference in resistance between foxtail millet and maize (**Figure 1A**).

### 3.3 Effects of nicosulfuron on the expression of *ALS* genes in foxtail millet and maize

**Figure 2:**
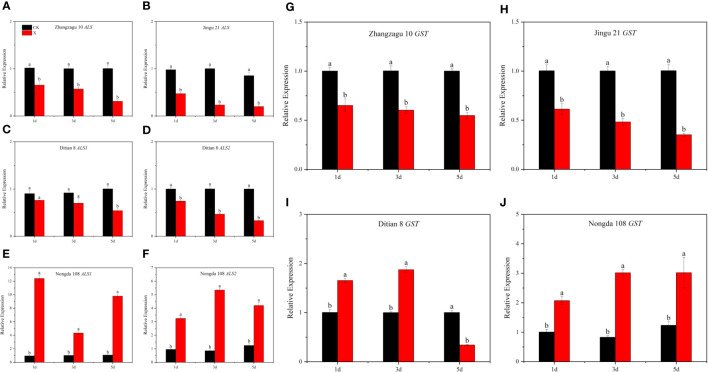
Effects of nicosulfuron on the expression of related genes in foxtail millet and maize at 5 d after spraying. **(A)** Zhangzagu 10 *ALS*, **(B)** Jingu 21 *ALS*, **(C)** Ditian 8 *ALS1*, **(D)** Nongda 108 *ALS1*, **(E)** Ditian 8 *ALS2*, **(F)** Nongda 108 *ALS2*, **(G)** Zhangzagu 10 *GST*, **(H)** Jingu 21 *GST*, **(I)** Ditian 8 *GST*, and **(J)** Nongda 108 *GST*. Data are presented as the mean ± SE (n = 6). Different letters indicate that the differences were statistically significant at *P* < 0.05.

After nicosulfuron treatment, the relative expression of the *ALS* gene in foxtail millet was downregulated at 1, 3, and 5 d. The relative expression levels of the *ALS* gene in Zhangzagu 10 and Jingu 21 at 5 d were downregulated by 69.07% and 76.64%, respectively, compared with those in the control. Compared with those in the control, the relative expression levels of *ALS1* and *ALS2* genes in Ditian 8 at 5 d were significantly downregulated by 46.01% and 66.95%, respectively. The relative expression levels of *ALS1* and *ALS2* genes in Nongda 108 were significantly upregulated by 817.92% and 238.72%, respectively, at 5 d compared with those in the control (**Figures 2A–F**).

### 3.4 Effects of nicosulfuron on the GST activity of foxtail millet and maize

Nicosulfuron treatment had different effects on GST activity in foxtail millet and maize (**Figure 1B**). Under the stress of the 1/8X nicosulfuron dose, the GST activity of Zhangzagu 10 and Jingu 21 decreased significantly by 26.70% and 20.81%, respectively, when compared with that in the CK. The activity of GST in Ditian 8 first increased and then decreased with the increase in nicosulfuron dosage. The GST activity of Nongda 108 increased with nicosulfuron dosage and reached its maximum with the 2X treatment. Compared with that of CK, the GST activity of Nongda 108 significantly increased by 100.04%, and the differences among the treatments were significant.

### 3.5 Effects of nicosulfuron on *GST* gene expression in foxtail millet and maize

Nicosulfuron treatment significantly affected the expression level of the *GST* gene (**Figures 2G–J**). Under the recommended dose of nicosulfuron, compared with control, the relative expression of the *GST* gene in foxtail millet was significantly downregulated at 1, 3, and 5 d, and that in Zhangzagu 10 and Jingu 21 were significantly downregulated by 45.14% and 64.92% at 5 d, respectively. The relative expression of *GST* gene in Ditian 8 was significantly upregulated at 1 and 3 d and significantly downregulated at 5 d, which was significantly downregulated by 65.93% compared with that of the control. The relative expression of the *GST* gene in Nongda 108 was significantly upregulated after 1, 3, and 5 days, which was upregulated by 106.05%, 264.71%, and 144.11%, respectively, compared with that of the control.

### 3.6 Effects of nicosulfuron on CYP450 activity in foxtail millet and maize

After treatment with nicosulfuron, the CYP450 activity of foxtail millet and maize showed an upward trend (**Figure 1C**). Compared with the control, the CYP450 activity of Zhangzagu 10 significantly increased by 5.65%, 15.23%, 21.40%, and 36.89% under 1/8X, 1/4X, 1/2X, and 1X treatment, while Jingu 21 significantly increased by 28.10%, 37.24%, 67.90%, and 110.92%, respectively. The increase of CYP450 activity in Jingu 21 was greater than that in Zhangzagu 10, indicating that under different nicosulfuron stress conditions, CYP450 activity in Jingu 21 contributed to the detoxification of nicosulfuron more than in Zhangzagu 10, reducing the phytotoxicity of nicosulfuron to foxtail millet. Similarly, nicosulfuron stress promoted the continuous increase of CYP450 activity in maize, and under the same spraying treatment, the CYP450 activity of Nongda 108 was higher than that of Ditian 8.

### 3.7 Effects of nicosulfuron on the malonaldehyde (MDA) content in foxtail millet and maize

**Figure 3:**
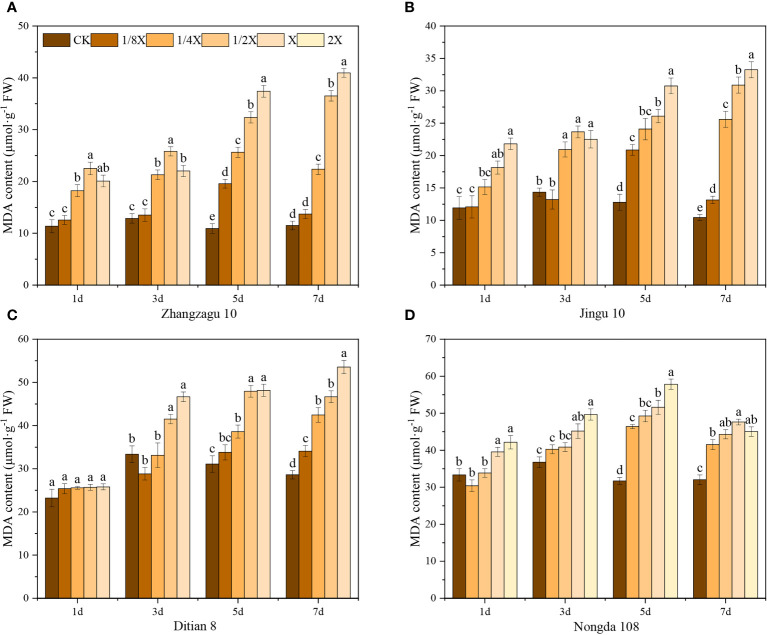
Effects of nicosulfuron on the MDA of foxtail millet and maize. **(A)** Zhangzagu 10, **(B)** Jingu 21, **(C)** Ditian 8, and **(D)** Nongda 108. Data are presented as the mean ± SE (n = 6). Different letters indicate statistically significant differences at *P* < 0.05.

Nicosulfuron treatments increased the MDA content in both foxtail millet and maize (**Figure 3**). The results showed that there was no significant difference MDA content in foxtail millet with the 1/8X dose when compared with the control after spraying with nicosulfuron for 3 d. Maize had similar results at higher doses, indicating that foxtail millet and maize were not sensitive to low doses of nicosulfuron at the initial stages of herbicide spraying. With the extension of time after application, the MDA contents of Zhangzagu 10 and Jingu 21 first increased and then decreased under the 1/4X and 1/8X doses, and reached their maximum levels after 5 d with significant increases of 134.75% for Zhangzagu 10 and 63.24% for Jingu 21, compared with that in the control. Ditian 8 also had similar results under the 1/2X dose, while the resistant Nongda 108 showed similar results with all doses. These results indicate that foxtail millet and maize have the ability to alleviate the damage caused by low doses of nicosulfuron on plants 5 d after application. In contrast, under high doses of nicosulfuron treatment, there was no significant recovery.

### 3.8 Effects of nicosulfuron on the superoxide dismutase (SOD), peroxidase (POD) and catalase (CAT) activities in foxtail millet and maize

The SOD activity of foxtail millet and maize increased with an increase in nicosulfuron dose; the highest level of activity was reported with the maximum dose (**Figure 1D**). With the recommended dose, the SOD activities in Zhangzagu 10, Jingu 21, Ditian 8, and Nongda 108 increased by 260.40, 362.72, 44.94, and 20.19%, respectively, compared with that in the control. Similarly, nicosulfuron treatments resulted in increased POD activity in foxtail millet and maize (**Figure 1E**). With the recommended dose, POD activities in Zhangzagu 10, Jingu 21, Ditian 8, and Nongda 108 increased by 195.42, 67.22, 68.17, and 24.56%, respectively, when compared with that in the control. However, the CAT activity in foxtail millet showed a different activity pattern (**Figure 1F**). As the nicosulfuron dose increased, the CAT activity of foxtail millet first increased and then decreased, and the minimum value was observed with the 1X dose. Compared with that of the control, the CAT activities of Zhangzagu 10 and Jingu 21 decreased by 7.48% and 7.57% after 1X nicosulfuron exposure, but the difference was not significant. The CAT activity of maize followed the same pattern as that of SOD and POD activities. The maximum CAT activity occurred with the maximum dose, and when compared with that in the control, it significantly increased by 45.77% in Zhangzagu 10 and 35.60% in Jingu 21.

## 4 Discussion

The safety of herbicides in crops can be proved by analyzing the agronomic traits (height, leaf area, biomass, yield, etc.) (Yuan et al., 2013). Herbicides such as pyrazosulfuron-methyl, tribenuron-methyl, fluroxypyr have been shown to be unsafe for foxtail millet through agronomic traits (Ning et al., 2015; Guo et al., 2018; Ma et al., 2021). In this study, nicosulfuron spray doses of ≥1/4X significantly decreased the yield of Ditian 8 and Zhangzagu 10 when compared with the control. While nicosulfuron doses of ≥ 1/8X significantly decreased the yield of Jingu 21, the yield of Nongda 108 was not significantly different from that of the control (**Tables 3**, **4**). Grain yield is affected by ear length, ear weight and grain weight, which together determine the yield. Herbicides, including nicosulfuron, can affect these components to different extents (Ning et al., 2015). In this study, the ear length, ear weight, and 1000-grain weight of foxtail millet decreased as the nicosulfuron dose increased, and the Zhangzagu 10 and Jingu 21 plants died with the recommended dose (**Tables 3**, **4**), indicating that these doses exceeded the regulatory capacity of these plants. Our previous study showed that nicosulfuron inhibited plant height, leaf area and photosynthetic capacity of foxtail millet and sensitive maize (Gao et al., 2021). But more than 90 percent of crop yield comes from photosynthesis (Yuan et al., 2013). Therefore, we believe that nicosulfuron causes drug damage to foxtail millet and maize, inhibits growth, affects photosynthetic capacity, and then affects ear type and ear weight, and ultimately leads to yield reduction.

Nicosulfuron can inhibit ALS activity, resulting in plant death caused by a deficiency of branched-chain amino acids (Saika et al., 2014; Rey-Caballero et al., 2017; Shah et al., 2022). Huang et al. (2019) found that the ALS activity inhibition rate (30–50%) of resistant *Amaranthus retroflexus* L. populations was significantly lower than that of sensitive populations (80%) under nicosulfuron treatment. Lan et al. (2022) proved that the ALS activity of Japanese brome (*Bromus japonicus* Thunb.) with a high level of resistance was significantly higher than that of the sensitive type after spraying flucarbazone-sodium. In our study, we found that nicosulfuron had an inhibitory effect on the ALS activity of foxtail millet and sensitive maize (Ditian 8), and the inhibitory effect increased with the increase in dose, As expected, the resistant variety Nongda 108 maintained higher ALS activity and exhibited less sensitivity to nicosulfuron compared to other crops (**Figure 1A**). The sensitivity of ALS to nicosulfuron was Jingu 21 > Zhangzagu 10 > Ditian 8 > Nongda 108. The reason may be that under the stress of nicosulfuron, Nongda 108 can achieve the physiological metabolism balance of ALS through self-regulation, thus alleviating the damage caused by herbicide spraying. In addition, we selected *ALS* genes to analyze their expression in foxtail millet and maize treated with nicosulfuron. Furthemore, it has been found that pyruvate metabolism, and operate upstream of the *ALS* genes, can restore the resistance to herbicide stress in cotton (Thyssen et al., 2014). In our study, increased *ALS* gene expression was observed in nicosulfuron-tolerant maize (**Figures 2E, F**). This upregulation may be attributed to the activation of gene expression associated with pyruvate metabolism caused by herbicides. In conclusion, the change of ALS enzyme and related genes is an important reason for the difference of resistance between foxtail millet and maize to nicosulfuron.

With the in-depth study of the mechanisms of plant resistance to herbicides, NTSR mechanisms have been gradually recognized as important for plant resistance to ALS inhibitor herbicides (Yu et al., 2014). The first stage of metabolizing herbicides mainly involves P450s, which catalyze the hemoglobinase system. The enzyme system re-catalyzes the monooxygenation reaction involving NADPH, which reduces the toxicity of the herbicide, thereby reducing the damage to the plant (Jensen and Møller, 2010). Previous studies have shown that increasing the activity of cytochrome P450 can reduce the damage of nicosulfuron to crops (Owen et al., 2012; Mei et al., 2017; Choe and Williams, 2020). For example, rice plants exposed to atrazine showed a significant increase in CYP450 activity, with 1.7-fold and 5.9-fold increases in root and shoot CYP450 activity, respectively, resulting in good levels of plant protection (Tan et al., 2015). This study found that CYP450 activity in foxtail millet and maize was significantly higher than that of the control (**Figure 1C**), indicating that CYP450 was actively involved in the detoxification of nicosulfuron in foxtail millet and maize.

GSTs are play an important role in herbicide metabolism (Cummins et al., 2013; Singh et al., 2015), and studies have shown that the GST activity of resistant plants is significantly higher than that of sensitive plants (Shopova et al., 2021; Zhang et al., 2021b). Our study showed that GST activity increased with the increased of nicosulfuron dose in Nongda108 (**Figure 1B**), which may be due to Nongda 108 could reduce the toxicity of nicosulfuron by catalyzing the condensation reaction of glutathione (GSH) with heterologous compounds and transporting metabolites to vacuoles or cell walls through transporters. However, even at low doses, the GST of Zhangzagu 10 and Jingu 21 still resulted in irreparable damage and could not be detoxified normally (**Figure 1B**). The results thus show that GST can improve the ability of plants to resist adverse environmental conditions and will be essential for the cultivation of new varieties of stress-resistant crops. In addition, we determined the *GST* gene expression in foxtail millet and maize. Moreover, studies have shown that the accumulation of H_2_O_2_ triggers the expression of *GST* and stimulates the activity of GST enzyme, which is an important reason for the resistance of maize to halosulfuron-methyl (Levine et al., 1994; Pan et al., 2017). Similar results were observed in nicosulfuron-tolerant maize in our study, and this may explain the reason why the GST activity in tolerant maize leaves increased after nicosulfuron exposure. In summary, GST activity and gene expression are important reasons for the difference in resistance to nicosulfuron between foxtail millet and maize.

Herbicides destroys the balance between reactive oxygen species (ROS) production and remova and induces lipid peroxidation (MDA as a result of lipid peroxidation reaction) (Jalal et al., 2021), which has been demonstrated in maize (Panfili et al., 2019), wheat (Bao et al., 2020), and soybean (Li et al., 2020). Our results showed an increase in MDA content in the nicosulfuron-treated samples (**Figure 3**), indicated that the cell integrity of the foxtail millet and maize leaves was compromised, which was consistent with previous studies. However, with the prolongation of treatment time, the higher resistance of Nongda 108 meant that it was protected from nicosulfuron membrane lipid peroxidation, while Ditian 8, Zhangzagu 10, and Jingu 21 could only alleviate low-dose nicosulfuron-induced membrane damage to a certain degree (**Figure 3**). Previous studies have shown that herbicide stress on peanut, soybean, wheat, foxtail millet and maize increase antioxidant capacity and protect plants from damage (Jiang and Yang, 2009; Radwan, 2012; Jiang et al., 2016; Guo et al., 2018; Guan et al., 2020; Wu et al., 2022). SOD, POD, and CAT are important protective enzymes (Dazy et al., 2009). SOD is the first line of defense against ROS damage, catalyzing the dismutation of superoxide radical to O_2_ and H_2_O_2_. POD and CAT are the key enzymes for eliminating H_2_O_2_. The enzyme POD, which utilizes phenolic compounds as substrates for the decomposition of H_2_O_2_, is widely distributed across various plant tissues (Yang et al., 2008). Our study revealed an important stimulation of SOD and POD activity after nicosulfuron application (**Figures 1D, E**), which may be a response to the accumulation of ROS and especially superoxide anion. This adaptive mechanism aimed to maintain normal growth despite the herbicide stress. However, the CAT of foxtail millet showed a trend of increasing first and then decreasing with the increase of nicosulfuron dosage (**Figure 1F**), which may be due to the differences in regulation and coordination mechanisms, response times, and degree of change of each index in the antioxidant enzyme system (Zhao et al., 2020). Similarly, this also indicated that Zhangzagu 10 and Jingu 21 could not respond to the nicosulfuron-induced oxidative stress through the antioxidant enzyme system alone.

## 5 Conclusions

The results of this study demonstrate that foxtail millet is less resistant to nicosulfuron than maize, even in the same crop (maize), different varieties had different resistance to nicosulfuron. The differences in resistance were found to be associated with reduced target enzyme activity, detoxification enzyme activity, and gene expression. These findings offer valuable insights for exploring the TSR and NTSR mechanisms in foxtail millet under herbicide stress and provides theoretical basis for future research of develop foxtail millet germplasm with diverse herbicide resistance traits.

## Data availability statement

The original contributions presented in the study are included in the article/**Supplementary material**. Further inquiries can be directed to the corresponding authors.

## Author contributions

BL: Data curation, Formal analysis, Visualization, Writing – original draft. RM: Formal analysis, Writing – review & editing. YRW: Data curation, Resources, Writing – review & editing. WX: Visualization, Writing – review & editing. YM: Resources, Writing – review & editing. PG: Data curation, Writing – review & editing. JR: Writing – review & editing. LZ: Writing – review & editing. ZZ: Conceptualization, Writing – review & editing. GF: Conceptualization, Writing – review & editing. YYW: Supervision, Writing – review & editing. XY: Conceptualization, Funding acquisition, Methodology, Project administration, Supervision, Writing – review & editing.
