# Supplementary figures and images for: Distinctive physiological and molecular responses of foxtail millet and maize to nicosulfuron
Source: Front Plant Sci. 2024 Jan 16;14:1308584. doi: 10.3389/fpls.2023.1308584 (PMC10824897; doi:10.3389/fpls.2023.1308584)

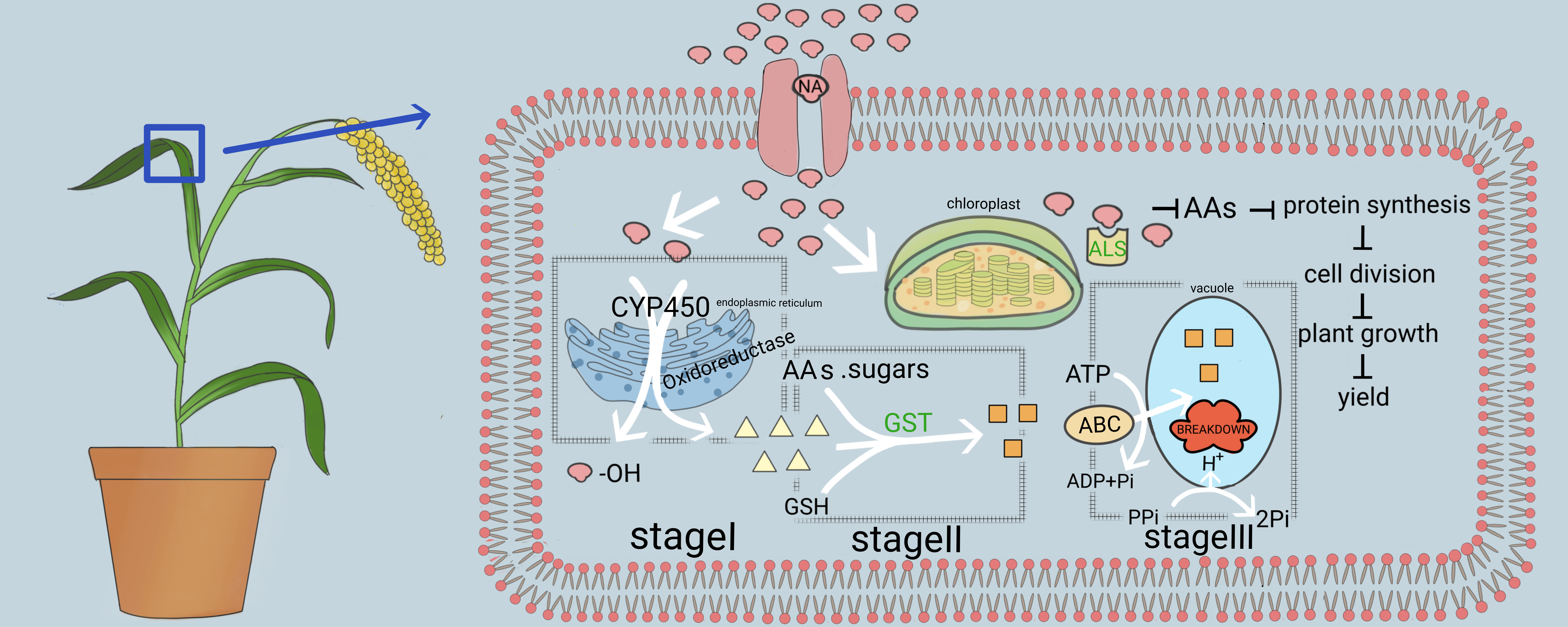

Supplement: Supplementary file 1 [file Image_1.png]
